# Supplementary figures and images for: Nonequilibrium brain dynamics elicited as the origin of perturbative complexity
Source: PLoS Comput Biol. 2025 Jun 6;21(6):e1013150. doi: 10.1371/journal.pcbi.1013150 (PMC12173227; doi:10.1371/journal.pcbi.1013150)

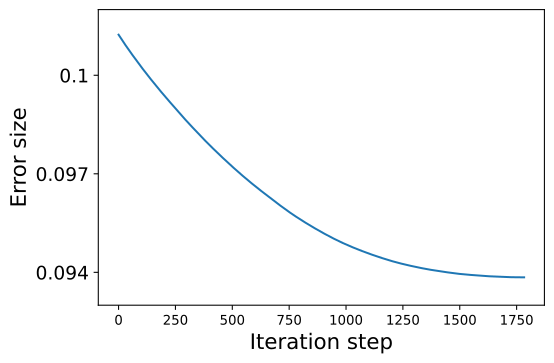

Supplement: S1 Fig — In an iterative process the time-lagged covariances of the simulated time series are compared to those of the empirically retrieved time series. In the case of a positive error, i.e., the empirical value is higher than the simulated value, the weight of the gEC of that connection is increased to create a stronger connection and consequently a higher value in the time-lagged covariance. For a negative error the process is the same but reversed, the weight of the connection is decreased. The error shown in this graph is the error over all time-lagged covariances in the matrix of this model. (TIFF) [file pcbi.1013150.s001.tiff]

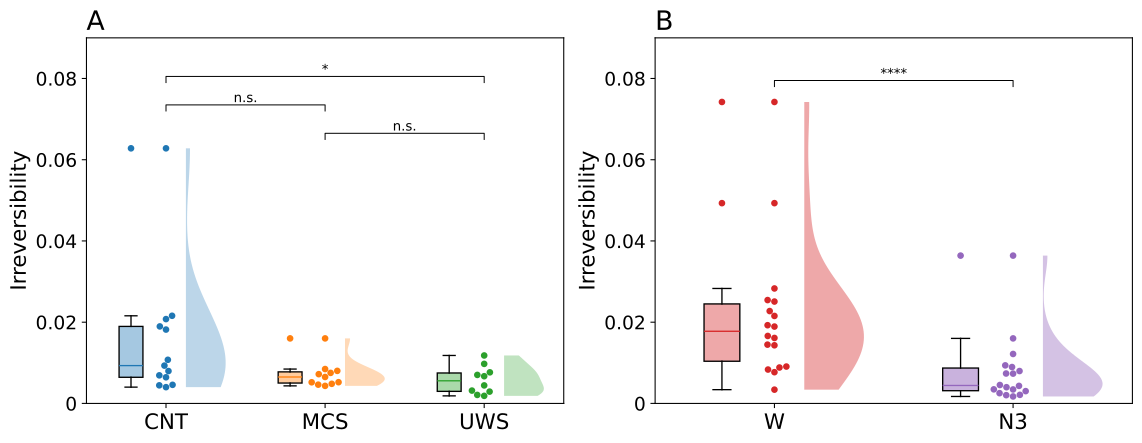

Supplement: S2 Fig — A) The irreversibility values for the DoC dataset. B) The irreversibility values for the sleep dataset. CNT - control, MCS - Minimally Conscious State, UWS - Unresponsive Wakefulness State, W – wakefulness, N3 – deep sleep state. (TIFF) [file pcbi.1013150.s002.tiff]

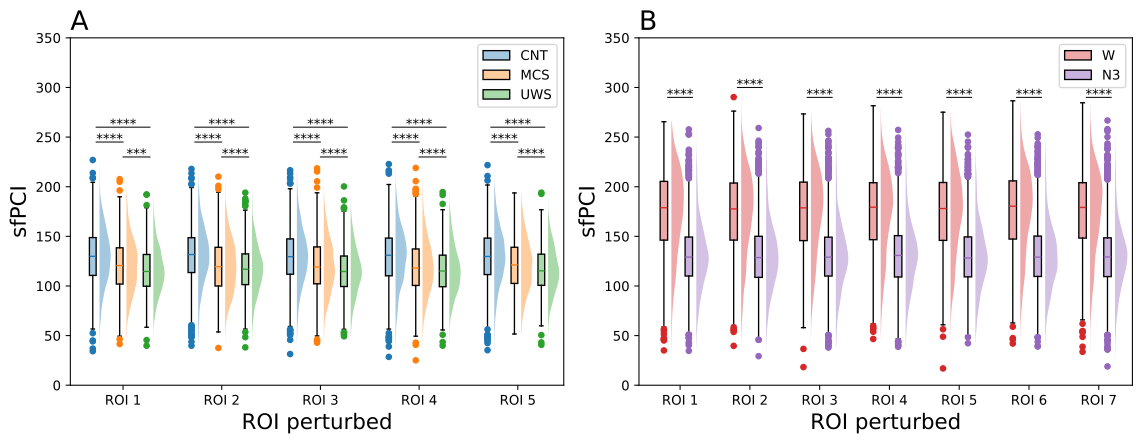

Supplement: S3 Fig — A perturbation of each node was simulated 100 times, i.e., each boxplot contains a number of datapoints equal to a 100 times the number of subjects in that category. In this figure, examples are depicted per node, showing that this trend where PCI is higher in CNT and W than in MCS, UWS and N3, is present regardless of the node perturbed. Less nodes are portrayed for the DoC dataset for visualization purposes. A) The sfPCI values for the DoC dataset. B) The sfPCI values for the sleep dataset. CNT - control, MCS - Minimally Conscious State, UWS - Unresponsive Wakefulness State, W – wakefulness, N3 – deep sleep state. (TIFF) [file pcbi.1013150.s003.tiff]
