# Supplementary material for: Nonequilibrium brain dynamics elicited as the origin of perturbative complexity
Source: PLoS Comput Biol. 2025 Jun 6;21(6):e1013150. doi: 10.1371/journal.pcbi.1013150 (PMC12173227; doi:10.1371/journal.pcbi.1013150)
Supplement: Attachment — Submitted filename: Response_reviewers_non-equilibrium_PCI_v2.docx [file pcbi.1013150.s005.docx]

**Response to the review of Manuscript PLOS Computational Biology (PCOMPBIOL-D-24-02115): “Nonequilibrium brain dynamics elicited as the origin of perturbative complexity”**

We are very grateful to the reviewers for their constructive feedback. We have systematically addressed all the reviewers' comments and suggestions. We have included new supplementary material and made substantial changes to the main text of the manuscript. Most of these changes are also reproduced here for the sake of clarity. We hope that our changes have resulted in a revision that is considered suitable for publication.

**Reviewer #1:**

**This paper is one by of the leading groups in the field. They converge resting state fMRI in reduced consciousness (sleep, UWS) and converge them with their computational model to investigate the underlying thermodynamics. They observe that reduced states of consciousness show lower asymmetry in their effective connectivity, lower time irreversibility, and lower complexity. This is a well done paper which therefore only raises a few questions.**

We sincerely appreciate the positive reception of our work, and the constructive feedback that was provided. We have addressed your insights on the discrepancies, intelligibility, and the methodological explanations by rewriting and elaborating several parts of the manuscript to better communicate the work that was done and to spread our ideas to a wider audience.

**1. There seems to be a considerable discrepancy between authors summary and abstract with the PCI only being mentioned in the former but not the latter, that leaves me confused….**

We thank reviewer 1 for noticing this discrepancy. We have added the PCI to the abstract to make the overlap between the author’s summary and abstract more coherent. The abstract is now as follows:

*Assessing someone’s level of consciousness is a complex matter, and attempts have been made to aid clinicians in these assessments through metrics based on neuroimaging data. Many studies have empirically investigated measures related to the complexity elicited after the brain is stimulated to quantify the level of consciousness across different states. Here we hypothesized that the level of non-equilibrium dynamics of the unperturbed brain already contains the information needed to know how the system will react to an external stimulus. We created personalized whole-brain models fitted to resting state fMRI data recorded in participants in altered states of consciousness (e.g. deep sleep, disorders of consciousness) to infer the effective connections underlying their brain dynamics. We then measured the out-of-equilibrium nature of the unperturbed brain by evaluating the level of asymmetry of the inferred connectivity, the time irreversibility in each model and compared this with the elicited complexity generated after in silico perturbations, using a simulated fMRI-based version of the Perturbational Complexity Index, a measure that has been shown to distinguish different levels of consciousness in in vivo settings. Crucially, we found that states of consciousness involving lower arousal and/or lower awareness had a lower level of asymmetry in their effective connectivities, a lower level of irreversibility in their simulated dynamics, and a lower complexity compared to control subjects. We show that the asymmetry in the underlying connections drives the nonequilibrium state of the system and in turn the differences in complexity as a response to the external stimuli.*

**2. The introduction then starts right away with the PCI…as I Understand this is about PCI in the modelling rather than with TMS…**

Thank you for pointing this out, we have expanded this paragraph of the introduction to clarify that the PCI that we are using in this work is representative of the EEG-TMS that has been done before, but is in fact modeled here and not performed *in vivo*. We have also renamed this modelled version throughout the manuscript, as sfPCI (simulated fMRI-based PCI). The part of the paragraph explaining the PCI now reads as follows:

*An established approach to investigate this relationship was proposed by Massimini and colleagues, who assessed the perturbation-elicited changes in global brain activity during different states of consciousness, such as wakefulness, sleep, anaesthesia, and disorders of consciousness (DoC)* (1–3)*. Specifically, they computed the perturbational complexity index (PCI), which captures the significant differences in brain-wide spatiotemporal propagation of external stimulation, and they proposed it as an index of consciousness* (1)*. Importantly, the PCI had high accuracy in distinguishing different levels of consciousness and the results were replicated in multiple studies, showing its sensitivity and usefulness* (4–7)*. Although the PCI has been proven to be valuable and accurate, it requires an extensive clinical setup which allows for perturbation of the brain with Transcranial Magnetic Stimulation (TMS) while recording brain activity with electroencephalography (EEG). In 2022 the Explainable Consciousness Indicator (ECI)* (8) *was presented. In this article it was concluded that the different levels of awareness and arousal could already be distinguished between groups based on the unperturbed time series by using explainable deep learning models. In contrast to the work described in this paragraph, here we will be working with simulations. We will be using personalised models based on fMRI data to create in silico perturbations by changing the oscillations in the model, representing the TMS experiment, to calculate a simulated fMRI-based PCI (sfPCI).*

**3. They may want to better explain their notion of asymmetry in the introduction..not clear to me..and also the link from PCI to thermodynamics..**

Thank you for pointing out that these statements needed clarification. We now specifically explain what is meant by asymmetry in the context of our work and how PCI relates to thermodynamics in this theoretical framework. The parts of the introduction relating to these two statements now read as follow, for the asymmetry:

*Crucially, we introduced asymmetry in the models creating time hierarchy organization and breaking the detailed balance, causing there to be a state of nonequilibrium* (9)*. By incorporating the time asymmetry of brain dynamics, which reveal the generative underlying mechanism, asymmetry is introduced in the connections, named generative effective connectivity (gEC)* (9)*. Specifically, this asymmetry refers to the difference between the connections from region A to region B and from region B to region A, creating a bidirectional connectivity. The gEC is responsible for the balance of the causal interaction between brain regions and in turn the level of nonequilibrium in different states of consciousness.*

And for the link from PCI to thermodynamics, the paragraph now reads as follows:

*The main goal of the present work is to establish whether PCI, as a marker of consciousness in the human brain, can be associated with the dynamical nature of brain activity before being perturbed. We hypothesize that the level of nonequilibrium dynamics of the unperturbed system determines the response to an external stimulus and in turn the elicited complexity (PCI). To this end, we leverage recent works that proposed different theoretically-grounded approaches to measure the level of nonequilibrium at the whole-brain scale, such as entropy production* (10)*, violation of the Fluctuation Dissipation Theorem (FDT)*(11) *and temporal asymmetry of brain signals* (10–12)*. In particular, FDT provides a direct link between the state of the system and its response to a stimulus following the Onsager derivation* (13–15)*, equalizing the spontaneous fluctuations and the external perturbation* (11)*. Based on these thermodynamic concepts, previous works have empirically demonstrated that time asymmetry is reduced during deep sleep* (16) *and in DoC patients* (17)*. The work using FDT violations also showed that deep sleep and wakefulness are significantly different* (18)*. Here we focus on the mechanism behind this nonequilibrium nature of brain dynamics* (9) *and how this property can be related to PCI. By using simulated resting state data, perturbing the system, and looking at the recovery of its dynamics, we can directly relate the level of nonequilibrium to the sfPCI using one and the same simulation.*

**4. I am not sure: when converging the data with the model – do they take a structural connectivity matrix (DT) from the healthy usbjects, from a healthy data base or from the patients themselves? Even in the method this is not fully clear ot me, sorry**

Thank you for raising this point. We have clarified the fitting procedure further in the Overview subsection of the Results section. This part now reads as follows:

*Briefly, the fitting procedure starts with the Structural Connectivity (SC), which in this case is an averaged SC from a healthy group of subjects. We then start the simulations and update the weights of the model by comparing the simulated time-lagged covariances to their equivalents of the empirical data, i.e. the time-lagged covariances that were calculated for each subject individually (DoC dataset) and each state separately (sleep dataset). Figure 1C shows the comparisons between the newly simulated time-lagged covariance with the equivalent empirical values, and updates the weights of the model’s connections. The process is repeated until the error stagnates (Sup. Fig. 1) and we have generated an effective connectivity matrix, i.e. the gEC of the model.*

We have also expanded the Methods section on this subject. This part now reads as follows:

*A model is fitted to the empirical time-lagged covariances, depicted in Eq. 6, by iteratively adjusting the values of the connections in C_ij_ which are used in Eq. 2 and Eq. 3 to simulate time series. The fitting procedure starts by providing the model with the weights of the SC (obtained through DTI as mentioned above). The model is run and the simulated time-lagged covariances are calculated and compared to their empirical equivalent values. The weights of the model are then adjusted so that the simulated and empirical time-lagged covariances are as close as possible until the error between the two has stagnated (see SupFig. 1). The personalisation of the models was done by first creating grouped models. The time-lagged covariance matrix of each subject was calculated and averaged per group, yielding five grouped models: three grouped models for the DoC dataset (CNT, MCS, UWS) and two grouped models for the sleep dataset (W, N3). From there on each personalised model was created by taking the gEC of their respective grouped model as an initial step for the fitting process. Since the empirical time-lagged covariances come from the individual data per state, this results in personalised weights for each of the models, representing each subject in each state. An SC mask is used, meaning that all gECs in the end have connections in the same constellation but not with the same weights.*

**5. This should be clarified in the result part, I have not yet look at the method part..the fitting procedure of model and data is illustrated in figure 1 but not fully to me from that figure and the result text…**

Thank you for suggesting a clarification on this part. As written in point 4 for this reviewer, we have expanded the overview section of the Results, among other things, by adding a more elaborate explanation about the fitting procedure. We have also expanded the Methods about this subject, as can be found in point 4 as well. We have also included a Supplemental Figure (SupFig 1) in the paper that shows an example of the fitting procedure. The figure can be seen below as Review Figure 1, and in the paper as Supplemental Figure 1:

**Review Figure 1 –** *An example curve is shown of the fitting procedure for the gEC. In an iterative process the time-lagged covariances of the simulated time series are compared to those of the empirically retrieved time series. In the case of a positive error, i.e. the empirical value is higher than the simulated value, the weight of the gEC of that connection is increased to create a stronger connection and consequently a higher value in the time-lagged covariance. For a negative error the process is the same but reversed, the weight of the connection is decreased. The error shown in this graph is the error over all time-lagged covariances in the matrix of this model.*

**6. I am confused about the concept of generative effective connectivity…what is the difference to the standard concept of effective connectivity? Just that is now done in the model? Or does it show a substantial difference to the effective C in the data?**

We have clarified the concept in the Methods and the Results section and have included a figure in the Supplemental Figures. We trust that the changes we made after reading your comments in point 4 and 5 have addressed these points as well.

**7. The data in figure 3 do not look fully convincing to me.,.lots of outliers and large overlaps between HC and patients…….is that based on and the result of the personalizing the model of the individual gEC data?**

Yes, this is the result of personalizing the models, where each subject (in each state, for the sleep dataset) has a personalized gEC matrix. By using the unperturbed dynamics of the model we obtained 10 irreversibility values. We have used the same data here as the data used in Figure 3 in the manuscript. Review Figure 2 thus shows less data points after the removal of outliers, using a Median Absolute Deviation method to remove outliers, as the data is not normally distributed. There indeed exists an overlap between the healthy controls and the other categories, however, the control groups have a wider range of levels of irreversibility, making them distinguishable from the other groups.

**Review Figure 2 –** The same values are used as those for Figure 3 in the manuscript. Since the data is not normally distributed, we used the Median Absolute Deviation method to detect outliers based on > 2.5MAD (19). The results show that all distributions are significantly distinct from one another in the respective datasets when outliers are removed, using a Benjamini-Hochberg correction for multiple comparisons for the DOC dataset.

**8. They may want to show some empirical data of the group comparison…this will tell us how much the model is really better than the empirical data as the authros seem to claim**

Thank you for suggesting to show a direct comparison between the empirical and modelled data. These datasets contain fMRI data only, and no perturbations were performed when these data were gathered. This inhibits us from drawing a direct line from the empirical to the simulated PCI.

We have added a supplemental figure (Supplemental Figure 2) to the manuscript, shown here as Review Figure 3, with the figure description from the manuscript. The differences in irreversibility are somewhat present in the empirical results already, however, they are more pronounced in the simulated values. Our computational approach allowed us to enhance the differences between the categories through data augmentation. By running multiple simulations per subject we were able to calculate multiple irreversibility values per subject and thus per category, resulting in a more distinct categorization.

We have also described this in the Discussion of the manuscript, as follows:

*It has been widely investigated how the dynamics of the human brain are constrained and supported by the structural connectome* (20–23)*. In this sense, whole-brain models provided a suitable and successful avenue to investigate how the function of the brain is shaped by its structure* (21,24–26)*. Following the same rationale, we used the generative capabilities of whole-brain models to investigate how the gEC shapes the nonequilibrium of the brain dynamics and how that changes between states of consciousness. Crucially, we found a positive correlation between gEC asymmetry and the level of nonequilibrium in brain dynamics at subject level, suggesting that the broken detailed balance in the generative space determines the level of nonequilibrium at the whole-brain level, as has been demonstrated in other biological systems* (27)*. Our results also showed that the level of nonequilibrium in brain dynamics obtained with different gEC’s significantly decreases as the state of consciousness decreases, aligning with empirical results. The results of using the INSIDEOUT framework on the empirical data described in this article can be found in Sup. Fig. 2. The tendency for UWS and deep sleep to have lower irreversibility values than CNT and wakefulness is there, but is more easily found in our simulated data due to the data augmentation. Our simulated results also show the distinction between MCS and CNT and between MCS and UWS.*

**Review Figure 3 *-*** *The INSIDEOUT framework is applied to the empirically retrieved time series of each subject in to calculate the irreversibility, resulting in one value per subject per state. A) The irreversibility values for the DoC dataset. B) The irreversibility values for the sleep dataset. CNT - control, MCS - Minimally Conscious State, UWS - Unresponsive Wakefulness State, W – wakefulness, N3 – deep sleep state*

**9. They need to mention and cite some other literature on consciousness like the Temporo-spatial approach by the group around Northoff which also focuses on brain dynamics in disorders of consciousness although in slightly distinct ways….cite their recent papers and book…**

Yes of course, thank you for suggesting to add the work from Georg Northoff and his group on the temporo-spatial framework regarding consciousness. We have referred to several of their works and related this to our work in the manuscript. The paragraph in the Discussion on theories of consciousness reads as follows:

*Several theories of consciousness have been created over the years, amongst which some well-known theories exist such as the Global Neuronal Workspace Theory (GNWT)*(28)*, the Integrated Information Theory (IIT)*(29)*, and the Temporo-spatial Theory of Consciousness (TTC)* (30–32)*. Since the PCI stems from IIT, our results fall in line with their ideas around compressibility of neural activity, sharing similar results with their empirical setup where normal consciousness results in less compressible activity. The TTC not being bound by integration, but relying on temporospatial dynamics as the basis of consciousness, draws an interesting line to our results presented here. They discuss the unperturbed activity as the context in which a perturbation and integration can take place* (33)*. Here the gEC creates the basis and the level of nonequilibrium can be seen as the context. The irreversibility can be quantified, creating a context for the sfPCI.*

**10. And discuss some of the theories of Consciousness like iIT, GNWT and TTC….given that they apply one key measure of the IIT, the pCI…/.**

We have discussed other theories in the same paragraph in which we discuss Northoff’s work, as can be found under point 9 of this reviewer. We trust this answers this point as well.

-------------------------

**Reviewer #2: PCOMPBIOL-D-24-02115: Nonequilibrium brain dynamics elicited as the origin of perturbative complexity**

**Comments to the authors:**

**In this study, the authors apparently reanalyzed previously acquired data to created personalized whole-brain models of effective connectivity fitted to rsfMRI data of control subjects, and subjects in deep sleep and others with MCS or UWS. They then determined the out-of-equilibrium nature of brain dynamics by evaluating the level of asymmetry and time irreversibility in each model and compared it with a simulated complexity as elicited by a perturbation (mimicking TMS in empirical data). They found that the level of asymmetry, irreversibility, and complexity at a resting state predicts the complexity of a response to perturbation.**

**This is an interesting study. The paper is well written, clear, convincing, and straightforward. However, I would need a little bit more precision to perfectly understand what was actually done, and to correct a few weaknesses that appear in the paper. My concerns are detailed hereafter.**

We thank you sincerely for the positive reception of our research, and the constructive feedback. We have addressed your concerns regarding the clarity on the use and description of the datasets, clarified our terminology in our manuscript, and elaborated the parts containing statistical analyses.

**- The authors refer to previous studies having described the PCI as indicative of the complexity of brain dynamics. Another index has been described, the ECI, which can be calculated on EEG data without the need for TMS. Two types of ECI exist, one evaluating the level of arousal (ECIaro), and the other the level of information segregation and integration (ECIawa). I would be interested in knowing how the authors relate their findings to this ECI. It might be interesting to discuss this.**

Thank you for suggesting to include ECI in this manuscript. We have referenced the index in the introduction, in the paragraph where we introduce the TMS-EEG PCI, as well as the Discussion. Below you can find the parts we have added to both parts of the manuscript.

In the Introduction:

*Although the PCI has been proven to be valuable and accurate, it requires an extensive clinical setup which allows for perturbation of the brain with Transcranial Magnetic Stimulation (TMS) while recording brain activity with electroencephalography (EEG). In 2022 the Explainable Consciousness Indicator (ECI)* (8) *was presented. In this article it was concluded that the different levels of awareness and arousal could already be distinguished between groups based on the unperturbed time series by using explainable deep learning models. In contrast to the work described in this paragraph, here we will be working with simulations.* *We will be using personalised models based on fMRI data to create in silico perturbations by changing the oscillations in the model, representing the TMS experiment, to calculate a simulated fMRI-based PCI (sfPCI).*

In the Discussion:

*They also note several strict criteria to which this TMS-EEG need to be upheld. Following the line of thought that the PCI is determined by the level of nonequilibrium, indicates that the TMS-EEG setup could be redundant, as was previously concluded in the work describing the ECI through their ECI^awa^ (ECI in awareness component) based on empirical resting state data* (8)*. However, in this work we have added the notion of the level of nonequilibrium being the necessary information in resting-state data.*

**- In the dataset obtained from patients with disorders of consciousness, it is not clear to me whether the distinction between MCS+ and MCS- patients was done.**

Thank you for raising this point. We have clarified that this distinction was not made. The dataset description around this topic now reads as follows:

*We used fMRI data from 21 patiens with DoC’s. 10 of those patients were diagnosed with UWS (3 female, mean age 38.02 ± std of 17.02 ) and the other 11 patients were diagnosed with MCS (4 female, mean age 40.85 ± std of 15.04). No distinction was made between MCS+ and MCS-.*

**- The authors should be cautious regarding the terminology they use. At several places, they mention ‘reduced consciousness’ or ‘higher levels of consciousness’. To me, this terminology is too vague, because it is very difficult to quantify the degree of reduction or enhancement. I think the terms ‘altered consciousness’, ‘absence of consciousness’ or ‘normal consciousness’ would be much more appropriate.**

Thank you for raising this point. We have changed the terminology in the manuscript. Where previously ‘reduced consciousness’ was used, we now use ‘altered consciousness’ with more elaborate descriptions to refer to the different states of altered consciousness. Where previously we used ‘higher levels of consciousness’ we now use ‘normal consciousness’ where necessary, or use an indication when referring to MCS as compared to UWS. We have changed this terminology throughout the manuscript and will therefore not reproduce these instances here.

**- The authors do not discuss the limitations of their study. One limitation is that they used data acquired from other studies and reanalyzed them. This should be made clear, and associated limitations (sample size was not dedicated to the present study, there might have been a risk of type II statistical error, particularly regarding the results reported in Figure 2A). There are probably other limitations related to their analysis pipeline, which should be addressed (see also the other concerns of this reviewer).**

Thank you for noticing the absence of a limitation section. We have added a paragraph discussing the limitations in the Discussion section, it can be found as the second paragraph. This paragraph reads as follows:

*The work presented here should be considered within certain limitations. Firstly, all models were fitted from the same structural connectivity matrix, gathered from healthy subjects. Considering the DoC patients can have significant brain damage, this might influence the way these subjects are represented in our models. Secondly, the AAL parcellation has 90 regions, which is rather low considering that many parcellations used in computational neuroscience nowadays can often have 200 up to >1000 regions. Perturbational research might be more refined when using these parcellations, but computationally speaking (and coming from an environmentally sustainable point of view), these works are much heavier. Thirdly, the number of subjects in the datasets, in particular the DoC dataset, is rather low. This restricts us from dividing the MCS patient group into MCS- and MCS+ and gives us a less powerful conclusion altogether. The current research is restricted in several ways but these could be interesting follow up on in future research. A larger dataset could help in investigating how other factors come into play, such as age of subjects, the time since injury, and a distinction between MCS+ and MCS-.*

**- The brain was parcellated into 90 ROIs. This is a quite low number (some studies report parcellation into more the 1000 ROIs). Please explain this choice.**

In this study we chose to use 90 ROIs. This is because of the computational power and cost. Running models with 1000 ROIs takes an incredible amount of computational power, memory, and time. For example, to run the model with 90ROIs we need to fit the model, and run the model to simulate the time series. The fitting itself requires a calculation of the time-lagged covariances between all regions, in this case 90*90 = 1800 instances, whereas with 1000ROIs you need 1000*1000 = 10^6 instances. There is a slight adjustment of the weights, and these covariances need to be recalculated, often requiring > 1000 steps until the error stagnates. The same holds for simulating time series, where 90 nodes influencing each other is computationally much lower than 1000 nodes. The second involves time.

**- The authors mention having used the Benjamini-Hochberg correction for multiple comparisons. This correction is usually used to avoid identifying too many false positives (which is the case with the highly conservative Bonferroni correction). Please be more precise regarding the alpha threshold that was chosen for the BH correction, and the rationale behind this choice.**

We have more clearly explained what the alpha values are for a BH procedure. We have chosen the BH procedure to prevent finding too many false negatives, as it is slightly more lenient than the Bonferroni correction. We used the standard α value of 0.05 for the BH procedure, which is adjusted based on rank, which we now clarify in the Methods. This paragraph from the Methods now reads as follows:

*To test whether two distributions are significantly distinct from one another we used a permutation based ranksum t-test with 10,000 permutations. To correct for multiple comparisons, we used a Benjamini-Hochberg procedure, where the significance threshold (α = 0.05) is adjusted for each p-value based on their rank in order of magnitude. The threshold is then determined by dividing the p-value by the number of tests performed and multiplying by its rank. For example, if there are three tests performed these significance thresholds are based on α = 0.05 / 3 = 0.0167, multiplied by their rank. For rank 1, rank 2, and rank 3 these would be α = 0.0167, α = 0.033, and α = 0.05, respectively.*

**- In the paragraph entitled ‘gEC asymmetry characterizes different states of reduced consciousness’, the authors first report results obtained in DoC, then in sleep, then again in DoC. Please reorder the way results are presented.**

Thank you for noticing the ordering of the results in this section. We have reordered and rewritten this section, it now reads as follows:

*For the Disorders of Consciousness (DoC) dataset the distribution of the models fitted to the CNT subjects has significantly higher asymmetry values than the distribution of models fitted to the UWS subjects (p = 0.0043, Cohen’s d = 1.34). Between the MCS and UWS subjects (p = 0.0564, Cohen’s d = 0.44) and between the MCS and CNT subjects (p = 0.0820, Cohen’s d = 0.65) no significant differences were found. The distribution of asymmetry values for the MCS does not have a significantly different distribution from the CNT and UWS distributions, but the average does fall between the averages of the CNT and UWS distributions.*

*For the sleep dataset the difference between the two states W and N3 was found to be significant (p < 0.001, Cohen’s d = 2.65). Taken together, these results suggest that models fitted to subjects residing in normal consciousness tend to have a higher asymmetry in the gEC than those in altered states of consciousness.*

**- Please describe more clearly how subjects were chosen in the respective datasets (all subjects? Matched controls?).**

We have added how subjects were chosen in the respective datasets where information was missing previously. For the DoC datasets the categories were matched to have similar distributions in age and sex, we have added this information to the description of the dataset:.

*We used fMRI data from 21 patietns with DoC’s. 10 of those patients were diagnosed with UWS (3 female, mean age 38.02 ± std of 17.02 ) and the other 11 patients were diagnosed with MCS (4 female, mean age 40.85 ± std of 15.04). No distinction was made between MCS+ and MCS-. fMRI data was collected from an additional 13 healthy control subjects (7 female, mean age 42.54 ± std of 13.64).*

In the sleep dataset the data shown in the two stages comes from the same subjects. In other words, we have the Wakefulness stage and the deep sleep state (N3) for the 18 subjects included in this dataset. The dataset descriptions regarding selection now read as follows, for the DoC dataset:

*The patients' relatives gave their formal written informed consent for their familiar to participate, and all investigations were performed according to the Declaration of Helsinki and the French regulations.*

for the sleep dataset:

*Written informed consent was obtained, and the study was approved by the ethics committee of the Faculty of Medicine at the Goethe University of Frankfurt, Germany. The data used in this work comes from a larger database. The participants included in this work were those that reached all four polysomnographic sleep stages.*

and for the Structural Connectivity:

*Briefly, a SC matrix was obtained for each subject (n = 16) by using tractography algorithms to Diffusion Tensor Imaging, following a previously described methodology* (34)*. Participants were healthy and right-handed, and recruited online at Aarhus University in Denmark. Subjects that had a current diagnosis or a history of psychiatric or neurological disorders were not included.*

**- Please briefly describe how the EEG data (I presume acquired during fMRI acquisition in the sleep dataset) were cleaned before being assessed by expert for sleep stage classification.**

We have expanded the part of the Methods describing the Sleep and Wakefulness dataset, specifically the part on EEG data acquisition and polysomnography. This part of the Methods now reads as follows:

*Simultaneously to acquiring the fMRI data, polysomnography (PSG) was performed by recording EEG, EMG, ECG, EOG, pulse oximetry, and respiration. EEG was recorded using a cap (modified BrainCapMR, Easycap, Herrsching, Germany) with 30 channels, where the FCz electrode was used as reference. The sampling rate of the EEG was 5 kHz, a low-pass filter was applied at 250 Hz and a high-pass filter at 0.016 Hz. MRI and pulse artefact correction were applied based on the average artefact subtraction method* (35) *in Vision Analyzer2 (Brain Products, Germany), followed by objective (CBC parameters, Vision Analyzer) ICA-based rejection of residual artefact-laden components after subtracting the average artefact, resulting in EEG with a sampling rate of 250 Hz. EMG was collected with chin and tibial derivations, while the ECG and EOG were recorded bipolarly at a sampling rate of 5 kHz with a low-pass filter at 1 kHz. Pulse oximetry was collected using the Trio scanner, at a sampling rate of 50 Hz, and respiration was recorded with MR-compatible devices (BrainAmp MR+, BrainAmp ExG; Brain Products, Gilching, Germany). Participants were instructed to lie still in the scanner with their eyes closed and relax. Sleep classification was performed by a sleep expert based on the EEG recordings in accordance with the American Academy of Sleep Medicine criteria (2007)* (36)*.*

**- I personally do not understand the role of DTI in the analysis that was performed for this study. Please explain.**

Thank you for pointing out that this needed clarification. We have rewritten and expanded the Methods section on the Structural Connectivity and the generative Effective connectivity. The subsection on Structural Connectivity now reads as follows:

*We used the SC consisting of 90 ROI’s as presented in a previous study, for a more detailed description please refer to* (37)*, described in the section ‘DWI data collection and processing’. This dataset, and the SC gathered from it, are separate from the previously described datasets on DoC and sleep. Briefly, a SC matrix was obtained for each subject (n = 16) by using tractography algorithms to Diffusion Tensor Imaging, following a previously described methodology* (34)*. The connection between regions C_ij_ was calculated as the proportion of sampled fibres in all voxels in region i that reach any voxel in region j. DTI does not portray directionality of these fibres, therefore the average was taken between C_ij_ and C_ji_ as the undirected connection between regions i and j. The average was taken over the 16 subjects to portray a SC matrix representing a healthy connectivity. In this work, we use this SC as a starting point from where the fitting procedure can begin. In other words, this SC is used as the base from which the fitting procedure starts. It is also used as a mask during fitting, determining where connections can exist in the model, and where not, regardless of the weight of those connections.*

And on the generative Effective Connectivity:

*Each subject’s data was used to fit a generative Effective Connectivity (gEC) to create personalised models by using the linear approximation of the Hopf model* (38)*, depicted in Figure 1C. All data were band-pass filtered between 0.04-0.07 Hz before fitting.* *A model is fitted to the empirical time-lagged covariances, depicted in Eq. 6, by iteratively adjusting the values of the connections in C_ij_ which are used in Eq. 2 and Eq. 3 to simulate time series. The fitting procedure starts by providing the model with the weights of the SC (obtained through DTI as mentioned above). The model is run and the simulated time-lagged covariances are calculated and compared to their empirical equivalent values. The weights of the model are then adjusted so that the simulated and empirical time-lagged covariances are as close as possible until the error between the two has stagnated (see SupFig. 1). The personalisation of the models was done by first creating grouped models. The time-lagged covariance matrix of each subject was calculated and averaged per group, yielding five grouped models: three grouped models for the DoC dataset (CNT, MCS, UWS) and two grouped models for the sleep dataset (W, N3). From there on each personalised model was created by taking the gEC of their respective grouped model as an initial step for the fitting process. Since the empirical time-lagged covariances come from the individual data per state, this results in personalised weights for each of the models, representing each subject in each state. An SC mask is used, meaning that all gECs in the end have connections in the same constellation but not with the same weights.*

**- Regarding the Cohen’s d analysis, I would like to know what the authors considered a small, medium, or large effect size. This would allow having a better idea of the risk of type II statistical error.**

Thank you for pointing out that this was missing from the manuscript. We have added an explanation on what was considered the cutoff value for each of these descriptors. We have added this in the Methods section, and this reads as follows:

*To calculate and interpret the effect size of the difference between two distributions we used Cohen’s d effect size* (39)*. To describe effect size we use the descriptors small, medium, and large, described by Sullivan and Fein* (39) *by using cutoff values of 0.2, 0.5, and 0.8, respectively. We adjusted the effect sizes where needed when dealing with smaller sample sizes (N < 50) to correct for possible instability in small sample sizes.*

------------------------

**Reviewer #3: I thank the editor for the opportunity to review this manuscript regarding the nature of how brain dynamics is related to perturbative complexity in various states of consciousness. The manuscript, combining empirical and model-based approaches, shows that level of non-equilibrium dynamics at rest can predict how the modelled brain would react to a perturbation. This is an interesting idea with potential clinical applications. However, the use of the stPCI in fMRI data to disentangle states of consciousness has not been validated, which is important given that the temporal and spatial resolution of EEG (to which the stPCI is usually applied) is very different from fMRI. Furthermore, the manuscript poses bold statements regarding the causal relation between nonequilibrium brain dynamics and perturbative complexity, while merely correlations are used to support this claim and potentially many unknown or untested lurking variables could mediate the reported correlation.**

**Before giving some more concrete suggestions, I would like to this this opportunity to share some more general observations about how this work is presented. First, I’d like to ask the authors to revisit the statement that that clinicians must rely on behaviour rather than quantifiable metrics. Clinical assessments such as the CRS-R are designed to be quantifiable, rather than the clinical consensus diagnosis which is indeed associated with high levels of misdiagnosis (i.e., about 40%). If the authors refer to patients possessing covert consciousness instead, then it is true that neuroimaging assessments are useful (and recommended in the EU and USA) to complement the diagnosis. However, typically these auxiliary assessments are being validated on the behavioural assessment of patients – hence they are important and still the gold standard. I would therefore like to ask the authors for a clarification on this point, or to take caution when suggesting that neuroimaging-based measures would be “better” to objectify levels of consciousness than clinical assessment.**

We would like to thank reviewer 3 for sharing their thoughts on the claims we made in the previous version of the manuscript. We really appreciate their perspective, and have taken them into consideration and into writing. We have adjusted and narrowed our claims down throughout the article. We are more careful in statements regarding the clinical side of this story and have changed our conclusion as such. We have changed wording, making the tone less bold, and we state that the current results can help compliment current protocols rather than overrule clinicians and their work.

**Related to this, I believe that the implications of the current work are more modest than portrayed in the current version of the manuscript. Statements like “Through our models we demonstrated that the asymmetry in the generative underlying connections is causing the emergent nonequilibrium dynamics and, at the same time, the differences in the complexity elicited by perturbations measured with PCI.”, “Following the line of thought that the PCI is determined by the level of nonequilibrium, indicates that the TMS-EEG setup could be redundant.” And “Additionally, it could reduce the need for costly experiments, enhance statistical reliability, and minimize potential ethical issues.” are in my opinion problematic, as (1) the use of the stPCI has not been validated with real data. Though this limitation is mentioned in lines 314-320, its consequences are not reflected in the interpretation of the results nor discussion. (2) the causality of these relationships has not been proven and can thus still only be assumed. (3) It is questionable which ethical issues would be resolved by utilizing this model as there is a large overlap between the different groups and more generally, I wonder how ethical it is to replace an in-vivo experiment with a (compared to the actual brain) simplified model of it. Though I do see merit in these types of computational approaches, I believe it is important to also be mindful about their limitations.**

Thank you for raising these points. We have adjusted the bold wording to a more careful and considerate phrasing. We have taken out our statement about solving the ethical issue around working with DoC patients after taking into account the points you raised. In future work, we would like to look at the direct link between the nonequilibrium dynamics and the empirical PCI values. This would allow us to investigate the potential translational impact of our *in silico* findings. We have reproduced the phrases where we have changed the tone of the claims we make.

From the Abstract:

*We conclude that the information to know how the brain will react to such stimulations could already be found in the unperturbed dynamics.*

From the Introduction:

*Through our models we show that the asymmetry in the generative underlying connections is causing the emergent nonequilibrium dynamics and, at the same time, has an effect on the differences in the complexity elicited by perturbations measured with sfPCI.*

From the Discussion:

*Leveraging the thermodynamical description of nonequilibrium in brain dynamics* (40,41) *and computational whole-brain modelling* (24,35)*, we quantified how the level of nonequilibrium of brain dynamics is related to different states of consciousness and, in turn, how these differences can have an influence on the brain’s capability to respond to an external stimulus measured by the sfPCI.*

*Following the line of thought that the PCI is determined by the level of nonequilibrium in silico, this could be an indication that the same holds for the empirical PCI and that the TMS-EEG setup could be redundant.*

*This leads us to the hypothesis that there would be a strong correlation between the empirical PCI and the metrics presented here. Importantly, future work should focus on investigating this relationship based on empirical measures of non-equilibrium dynamics and FDT approaches* (18,42) *with the empirical PCI.*

*Additionally, it has potential to aid in reducing the need for costly experiments and enhance statistical reliability.*

**More specifically, yet related to the points raised above, I have the following questions:**

**-The original/ empirical PCI is well-known for its diagnostic precision. In the current work it seems that there is a large overlap between the diagnostic groups and states of consciousness. The reader might wonder if this is true, or if the overlap seems so large because the stimulation of all regions is presented in one figure. Do different figures show different PCI’s? Based on the paragraph starting on line 30, it seems that the PCI is invariant to the perturbed region, but I fail to find the data supporting that statement.**

There is a slight difference intracategorically when perturbing different areas. The differences between groups, however, does not change the statistical significance.

We have added a Supplementary Figure (SupFig. 3) with examples of different perturbed nodes. We have reproduced this figure and its description here as Review Figure 4, as well as referred to this supplementary figure in the main text as follows:

*To calculate the sfPCI values for the simulations both the preperturbational and the postperturbational time series were used to calculate the sfPCI, based on the State Transition PCI* (43)*. A simulation was performed for each node for each of the models, which are depicted individually in the boxplots shown in Figure 4, meaning there are 90 PCI values depicted for each subject. Examples of perturbations for specific nodes can be found in Sup. Fig. 3.*

**Review Figure 4** - *The sfPCI was calculated on simulated perturbations. A perturbation of each node was simulated 100 times, i.e. each boxplot contains a number of datapoints equal to a 100 times the number of subjects in that category. In this figure, examples are depicted per node, showing that this trend where PCI is higher in CNT and W than in MCS, UWS and N3, is present regardless of the node perturbed. Less nodes are portrayed for the DoC dataset for visualization purposes. A) The sfPCI values for the DoC dataset. B) The sfPCI values for the sleep dataset. CNT - control, MCS - Minimally Conscious State, UWS - Unresponsive Wakefulness State, W – wakefulness, N3 – deep sleep state*

Since the sfPCI values for the DoC dataset are more overlapping like you mentioned, we have created a figure (Review Figure 5) on the p-values for that dataset comparing the different categories for all ROIs. Note that the y-axes are not the same for all boxplots. Regardless of the ROI and the overlap in values between categories, the distributions remain significantly different from one another, as can be seen in the figure below:

**Review Figure 5** – A 100 simulations were performed per ROI, per subject. The p-values of a Wilcoxon ranksum permutation test are reproduced here, comparing the distributions of two categories per ROI perturbed. In other words, each point in the figure represents a p-value where the distributions between categories are compared for the same ROI, meaning each boxplot portrays 90 datapoints (for the 90 ROIs). Each boxplot represents the comparison between two categories. The boxplots CNT vs MCS and CNT vs UWS belong to the y-axis on the left, whereas the boxplot MCS vs UWS belongs to the y-axis on the right. CNT – control, MCS – Minimally Conscious State, UWS – Unresponsive Wakefulness State.

**-Related to this, it might be better to not use “PCI” when describing the results, but rather something along the lines of “model-based fMRI PCI”**

Thank you for pointing out that it might be better to clarify this. We have reintroduced this as ‘the simulated fMRI-based PCI’, or sfPCI for short. We have changed the terminology for clarity, we have not put these instances here but they can be found throughout the manuscript. We now introduce the sfPCI in the Introduction, written in the last sentence of the first paragraph of the manuscript as follows:

*Although the PCI has been proven to be valuable and accurate, it requires an extensive clinical setup which allows for perturbation of the brain with Transcranial Magnetic Stimulation (TMS) while recording brain activity with electroencephalography (EEG). In 2022 the Explainable Consciousness Indicator (ECI)* (8) *was presented. In this article it was concluded that the different levels of awareness and arousal could already be distinguished between groups based on the unperturbed time series by using explainable deep learning models. In contrast to the work described in this paragraph, here we will be working with simulations. We will be using personalised models based on fMRI data to create in silico perturbations by changing the oscillations in the model, representing the TMS experiment, to calculate a simulated fMRI-based PCI (sfPCI).*

**-Do the authors have an explanation for why irreversibility and the PCI seems to be higher in the awake HC group than in the DoC HC group?**

The wakefulness group in the sleep dataset has had higher nonequilibrium values than other control groups, e.g. it had higher nonequilibrium values compared to the HCP dataset (11). Since the datasets were gathered in different institutions with different setups, different parameters, different TR’s, this can bias the models and is the reason that we do not make comparisons between datasets.

**-I understand that no real PCI data of these patients is available to cross-check the fMRI model-based PCI. However, I believe that patients visiting this clinical center receive several other assessments (e.g., FDG-PET and EEG) that are used to refine the clinical diagnosis. If that is true, where there any covert conscious patients in this cohort, and how did the model’s prediction behave in these patients?**

Unfortunately, this cohort does not include this data. However, it is a very interesting point to look into and we have added this to the limitations and future directions paragraph in the Discussion. This paragraph reads as follows:

*The work presented here should be considered within certain limitations. Firstly, all models were fitted from the same structural connectivity matrix, gathered from healthy subjects. Considering the DoC patients can have significant brain damage, this might influence the way these subjects are represented in our models. Secondly, the AAL parcellation has 90 regions, which is rather low considering that many parcellations used in computational neuroscience nowadays can often have 200 up to >1000 regions. Perturbational research might be more refined when using these parcellations, but computationally (and sustainably) speaking these works are much heavier. Thirdly, the number of subjects in the datasets, in particular the DoC dataset, is rather low. This restricted us from dividing the MCS patient group into MCS- and MCS+ and gives us a less powerful conclusion altogether. Lastly, there is no direct link made between the empirical PCI and the sfPCI presented in this work. The translation made between our computational setup and a biological setting are therefore assumptions, based within a larger story of connected empirical and computational works. The current research is restricted in several ways that could be interesting to follow up on in future research. A larger dataset could help in investigating how other factors come into play, such as the time since injury, and a distinction between MCS+ and MCS-. A follow-up study of that could be to see how our models predict the prognosis of these patients. Furthermore, a connection between the computational and empirical PCI’s would be an important step in giving our claims more foundation.*

**-Regarding the relations between the irreversibility, asymmetry, and PCI, I am also interested to know if there would be a group effect. The authors have tested this using an LME with the group as random effect. I wonder why they did also including other clinical/demographic variables as time since injury, age etc? I understand that the sample size is modest, yet some preliminary investigation in that direction could be worthwhile. Also, the description of the LME approach should probably be moved to the methods section.**

Thank you for your ideas on testing for group effects. This is an incredibly interesting direction to investigate, to see whether there is any effect based on other variables besides their brain activity and diagnoses. We have looked into these group effects based on age, sex, and etiology. We found they did not have a significant effect on the correlations presented in our work. As for the placement of the LME approach, we have moved the methods part of this description to the Methods, which reads as follows:

*To test whether two metrics are correlated we used Spearman’s correlation to check for monotonic correlations and a non-parametric permutation-based test with 10,000 permutations. Again, significance thresholds for the p-values were corrected through a Benjamini-Hochberg procedure.*

*Linear mixed effect (LME) models were created to check whether these correlations were not influenced by the states of the subjects. LME models were created with and without the states as random effects, and were checked to be significantly different or not from one another through a likelihood ratio test (LRT). For the LME models to check the influence of demographic factors, the ages of the subjects were binned to create a discretized variable for the random effect, while the other factors were already discrete. This model included as random effects the age, etiology of the disordered state, gender, and the diagnosis of the patients. It did not include the control subjects, since the etiology of the disordered state was included as one of the random effects.*

And rewritten the paragraph in the Results, which reads as follows:

*Taken together, our results indicate that higher asymmetry is associated with higher values of sfPCI and irreversibility. This leads us to state that there is a strong and significant correlation between these measures that was not previously reported on in this setup.*

*Linear mixed effect (LME) models were created and compared to check for group effects based on the states of the subjects for all correlations shown in Figures 5 and 6. The models showed this was not the case, meaning the correlations shown here do not appear to be influenced by the states of the subjects. More LME models were created to check for demographic factors as well, including age, etiology of the disordered state, and gender. Since this model includes etiology of the disordered states, it does not include the control subjects. Again, these models showed that the correlations shown here do not appear to be influenced by these demographics.*

**-I do not understand which 16 datasets were used to create the average SC. The HC used in the HC data consists of 13, the HC from the sleep data consist of 18, then there are the patients, but it is unclear which data is used (and why only a selection is used?) to create the SC.**

Thank you for raising this point. We have expanded the subsection Structural Connectivity in the Methods section clarifying where the SC comes from. This subsection now reads as follows:

*We used the SC consisting of 90 ROI’s as presented in a previous study, for a more detailed description please refer to* (37)*, described in the section ‘DWI data collection and processing’. This dataset, and the SC gathered from it, are separate from the previously described datasets on DoC and sleep.* *Briefly, a SC matrix was obtained for each subject (n = 16) by using tractography algorithms to Diffusion Tensor Imaging, following a previously described methodology* (34)*. Participants were healthy and right-handed, and recruited online at Aarhus University in Denmark. Subjects that had a current diagnosis or a history of psychiatric or neurological disorders were not included. The connection between regions C_ij_ was calculated as the proportion of sampled fibres in all voxels in region i that reach any voxel in region j. DTI does not portray directionality of these fibres, therefore the average was taken between C_ij_ and C_ji_ as the undirected connection between regions i and j. The average was taken over the 16 subjects to portray a SC matrix representing a healthy connectivity. In this work, we use this SC as a base to fit our models to. In other words, this SC is used as the base from which the fitting procedure starts. It is also used as a mask during fitting, determining where connections can exist in the model, and where not, regardless of the weight of those connections.*

**-Related to the previous point, if the SC is based in the HC alone, how can the authors infer that it is representative for DoC patients as well? One could for example imagine that DoC patients show relatively more local connectivity than HC, but this would then not (or less so) be reflected in the CS, and consequently not be considered in the analysis. This could perhaps lead to an underestimation of the different metrics (and an overestimation of group differences).**

Thank you for raising this point. Not having the structural connectivities for the patients is indeed a limitation of this study (as added in the limitations paragraph in the Discussion, presented here 3 points above). Since we generate Effective Connectivities we do individualize the weights of all connections in each model based on the functional data, giving us personalized simulated data. Even though the SC mask that we use comes from healthy subjects, the model is still representative of the damaged patient brain in that sense. If there are regions that have no correlation in their time-lagged covariances, this will show up in the gEC as a low weight, and vice versa for highly connected regions. The SC serves as a mask in that it gives all patients the same constellation of connections, but different weights. This is also the reason why we state that this asymmetry, these weights, are causing the differences in the irreversibility and the PCI, as nothing else is different between the models. We have expanded the role of the SC in the Methods, as presented in the previous point.

**-I recommend the authors to report the group mean and SD for each of the metrics presented in the results section.**

Thank you for your recommendation to include the standard deviations for all metrics in the Results. We have included them in the Results. These particular parts of the sections now read as follows, for the asymmetry:

*For the Disorders of Consciousness (DoC) dataset the distribution of the models fitted to the CNT subjects (M = 1053, SD = 217) has significantly higher asymmetry values (p = 0.0043, Cohen’s d = 1.34) than the distribution of models fitted to the UWS subjects (M = 650, SD = 329). Between the MCS (M = 831, SD = 383) and UWS subjects (p = 0.0564, Cohen’s d = 0.44) and between the MCS and CNT subjects (p = 0.0820, Cohen’s d = 0.65) no significant differences were found.*

*For the sleep dataset the difference between the two states W (M = 1161, SD = 125) and N3 (M = 714, SD = 181) was found to be significant (p < 0.001, Cohen’s d = 2.65).*

For the irreversibility:

*The difference between CNT (M = 0.0339, SD = 0.0364) and MCS (M = 0.0212, SD = 0.0215) was significant (p = 0.008, Cohen’s d = 0.419), as were the differences between MCS and UWS (M = 0.0138, SD = 0.013) distributions (p = 0.002, Cohen’s d = 0.412), and between the CNT and UWS distributions (p < 0.001, Cohen’s d = 0.704).*

*The difference between the irreversibility values of W (M = 0.0607, SD = 0.0588) and N3 (M = 0.0249, SD = 0.0241) were significant as well, where the altered state of consciousness again had a lower level of irreversibility (p < 0.001, Cohen’s d = 0.799), which can be observed in Figure 3B.*

For the sfPCI:

*Significant differences were found in all three comparisons, after Benjamini-Hochberg correction for multiple comparisons, namely the comparison between the MCS (M = 122, SD = 27) and the UWS (M = 116, SD = 22) group (p < 0.001, Cohen’s d = 0.250), the comparison between CNT (M = 130, SD = 28) and MCS (p < 0.001, Cohen’s d = 0.307), and the comparison between CNT and UWS (p < 0.001, Cohen’s d = 0.570).*

*This can be confirmed by looking at Figure 4B where this trend is more pronounced, where the values for the W (M = 173, SD = 41) and N3 (M = 131, SD = 33) states are portrayed (p < 0.001, Cohen’s d = 1.129).*

**-Terms like irreversibility could be introduced in more detail so that the paper becomes accessible to a broader readership.**

Thank you for raising this point. We have rewritten the paragraph on irreversibility and the breaking of detailed balance in the Introduction. The paragraph now reads as follows:

*The concepts of detailed balance and irreversibility from thermodynamics have been applied to assess a system's ability to exhibit dynamic fluctuations between different states. When a system is reversible, it can still change from one state to another, but it is also able to reverse back without net fluxes of energy or activity. Such a system is said to adhere to detailed balance and is in a state of equilibrium. When a system is said to violate detailed balance, it exhibits net fluxes of e.g. activity, between its configurations, thereby establishing an arrow of time and rendering the system’s state irreversible, i.e. a state of nonequilibrium (12). Studies of spontaneous brain activity in healthy subjects have demonstrated that the brain operates in such a nonequilibrium state* (41)*.*

1.	Casali AG, Gosseries O, Rosanova M, Boly M, Sarasso S, Casali KR, et al. A Theoretically Based Index of Consciousness Independent of Sensory Processing and Behavior. Sci Transl Med [Internet]. 2013 Aug 14 [cited 2024 Jun 19];5(198). Available from: https://www.science.org/doi/10.1126/scitranslmed.3006294

2.	Ferrarelli F, Massimini M, Sarasso S, Casali A, Riedner BA, Angelini G, et al. Breakdown in cortical effective connectivity during midazolam-induced loss of consciousness. Proc Natl Acad Sci. 2010 Feb 9;107(6):2681–6.

3.	Massimini M, Ferrarelli F, Huber R, Esser SK, Singh H, Tononi G. Breakdown of Cortical Effective Connectivity During Sleep. Science. 2005 Sep 30;309(5744):2228–32.

4.	Sarasso S, Rosanova M, Casali AG, Casarotto S, Fecchio M, Boly M, et al. Quantifying Cortical EEG Responses to TMS in (Un)consciousness. Clin EEG Neurosci. 2014 Jan;45(1):40–9.

5.	Sinitsyn DO, Poydasheva AG, Bakulin IS, Legostaeva LA, Iazeva EG, Sergeev DV, et al. Detecting the Potential for Consciousness in Unresponsive Patients Using the Perturbational Complexity Index. Brain Sci. 2020 Nov 27;10(12):917.

6.	Wang Y, Niu Z, Xia X, Bai Y, Liang Z, He J, et al. Application of Fast Perturbational Complexity Index to the Diagnosis and Prognosis for Disorders of Consciousness. IEEE Trans Neural Syst Rehabil Eng. 2022;30:509–18.

7.	Casarotto S, Comanducci A, Rosanova M, Sarasso S, Fecchio M, Napolitani M, et al. Stratification of unresponsive patients by an independently validated index of brain complexity. Ann Neurol. 2016 Nov;80(5):718–29.

8.	Lee M, Sanz LRD, Barra A, Wolff A, Nieminen JO, Boly M, et al. Quantifying arousal and awareness in altered states of consciousness using interpretable deep learning. Nat Commun. 2022 Feb 25;13(1):1064.

9.	Kringelbach ML, Perl YS, Tagliazucchi E, Deco G. Toward naturalistic neuroscience: Mechanisms underlying the flattening of brain hierarchy in movie-watching compared to rest and task. Sci Adv. 2023 Jan 13;9(2):eade6049.

10.	Sanz Perl Y, Bocaccio H, Pallavicini C, Pérez-Ipiña I, Laureys S, Laufs H, et al. Nonequilibrium brain dynamics as a signature of consciousness. Phys Rev E. 2021 Jul 28;104(1):014411.

11.	Deco G, Lynn CW, Sanz Perl Y, Kringelbach ML. Violations of the fluctuation-dissipation theorem reveal distinct nonequilibrium dynamics of brain states. Phys Rev E. 2023 Dec 26;108(6):064410.

12.	Lynn CW, Cornblath EJ, Papadopoulos L, Bertolero MA, Bassett DS. Broken detailed balance and entropy production in the human brain. Proc Natl Acad Sci. 2021 Nov 23;118(47):e2109889118.

13.	Crisanti A, Ritort F. Violation of the fluctuation-dissipation theorem in glassy systems: basic notions and the numerical evidence. J Phys Math Gen. 2003 May 30;36(21):R181–290.

14.	Onsager L. Reciprocal Relations in Irreversible Processes. I. Phys Rev. 1931 Feb 15;37(4):405–26.

15.	Onsager L. Reciprocal Relations in Irreversible Processes. II. Phys Rev. 1931 Dec 15;38(12):2265–79.

16.	Deco G, Sanz Perl Y, Bocaccio H, Tagliazucchi E, Kringelbach ML. The INSIDEOUT framework provides precise signatures of the balance of intrinsic and extrinsic dynamics in brain states. Commun Biol. 2022 Jun 10;5(1):572.

17.	G-Guzmán E, Perl YS, Vohryzek J, Escrichs A, Manasova D, Türker B, et al. The lack of temporal brain dynamics asymmetry as a signature of impaired consciousness states. Interface Focus. 2023 Jun 6;13(3):20220086.

18.	Monti JM, Perl YS, Tagliazucchi E, Kringelbach M, Deco G. The fluctuation-dissipation theorem and the discovery of distinctive off-equilibrium signatures of brain states [Internet]. 2024 [cited 2024 Sep 13]. Available from: http://biorxiv.org/lookup/doi/10.1101/2024.04.04.588056

19.	Leys C, Ley C, Klein O, Bernard P, Licata L. Detecting outliers: Do not use standard deviation around the mean, use absolute deviation around the median. J Exp Soc Psychol. 2013 Jul;49(4):764–6.

20.	Suárez LE, Markello RD, Betzel RF, Misic B. Linking Structure and Function in Macroscale Brain Networks. Trends Cogn Sci. 2020 Apr;24(4):302–15.

21.	Honey CJ, Kötter R, Breakspear M, Sporns O. Network structure of cerebral cortex shapes functional connectivity on multiple time scales. Proc Natl Acad Sci. 2007 Jun 12;104(24):10240–5.

22.	Honey CJ, Sporns O, Cammoun L, Gigandet X, Thiran JP, Meuli R, et al. Predicting human resting-state functional connectivity from structural connectivity. Proc Natl Acad Sci. 2009 Feb 10;106(6):2035–40.

23.	Luppi AI, Singleton SP, Hansen JY, Jamison KW, Bzdok D, Kuceyeski A, et al. Contributions of network structure, chemoarchitecture and diagnostic categories to transitions between cognitive topographies. Nat Biomed Eng [Internet]. 2024 Aug 5 [cited 2024 Sep 13]; Available from: https://www.nature.com/articles/s41551-024-01242-2

24.	Deco G, Kringelbach ML, Jirsa VK, Ritter P. The dynamics of resting fluctuations in the brain: metastability and its dynamical cortical core. Sci Rep. 2017 Jun 8;7(1):3095.

25.	Cabral J, Kringelbach ML, Deco G. Functional connectivity dynamically evolves on multiple time-scales over a static structural connectome: Models and mechanisms. NeuroImage. 2017 Oct;160:84–96.

26.	Deco G, Ponce-Alvarez A, Hagmann P, Romani GL, Mantini D, Corbetta M. How Local Excitation-Inhibition Ratio Impacts the Whole Brain Dynamics. J Neurosci. 2014 Jun 4;34(23):7886–98.

27.	Battle C, Broedersz CP, Fakhri N, Geyer VF, Howard J, Schmidt CF, et al. Broken detailed balance at mesoscopic scales in active biological systems. Science. 2016 Apr 29;352(6285):604–7.

28.	Dehaene S, Kerszberg M, Changeux JP. A neuronal model of a global workspace in effortful cognitive tasks.

29.	Farisco M, Changeux JP. About the compatibility between the perturbational complexity index and the global neuronal workspace theory of consciousness. Neurosci Conscious. 2023 Jun 19;2023(1):niad016.

30.	Northoff G, Huang Z. How do the brain’s time and space mediate consciousness and its different dimensions? Temporo-spatial theory of consciousness (TTC). Neurosci Biobehav Rev. 2017 Sep;80:630–45.

31.	Northoff G, Wainio-Theberge S, Evers K. Is temporo-spatial dynamics the “common currency” of brain and mind? In Quest of “Spatiotemporal Neuroscience”. Phys Life Rev. 2020 Jul;33:34–54.

32.	Northoff G, Buccellato A, Zilio F. Connecting brain and mind through temporo-spatial dynamics: Towards a theory of common currency. Phys Life Rev. 2025 Mar;52:29–43.

33.	Northoff G, Zilio F. From Shorter to Longer Timescales: Converging Integrated Information Theory (IIT) with the Temporo-Spatial Theory of Consciousness (TTC). Entropy. 2022 Feb 13;24(2):270.

34.	Cabral J, Kringelbach M, Deco G. Functional Graph Alterations in Schizophrenia: A Result from a Global Anatomic Decoupling? Pharmacopsychiatry. 2012 May;45(S 01):S57–64.

35.	Breakspear M. Dynamic models of large-scale brain activity. Nat Neurosci. 2017 Mar;20(3):340–52.

36.	AASM. The AASM Manual for the Scoring of Sleep and Associated Events: Rules, Terminology and Technical Specfications. Westchester: American Academy of Sleep Medicine; 2007.

37.	Ipiña IP, Kehoe PD, Kringelbach M, Laufs H, Ibañez A, Deco G, et al. Modeling regional changes in dynamic stability during sleep and wakefulness. NeuroImage. 2020 Jul;215:116833.

38.	Ponce-Alvarez A, Deco G. The Hopf whole-brain model and its linear approximation. Sci Rep. 2024 Jan 31;14(1):2615.

39.	Sullivan GM, Feinn R. Using Effect Size—or Why the *P* Value Is Not Enough. J Grad Med Educ. 2012 Sep 1;4(3):279–82.

40.	Lynn CW, Bassett DS. The physics of brain network structure, function and control. Nat Rev Phys. 2019 Mar 27;1(5):318–32.

41.	Kringelbach ML, Sanz Perl Y, Deco G. The Thermodynamics of Mind. Trends Cogn Sci. 2024 Jun;28(6):568–81.

42.	Patow G, Monti J, Acero-Pousa I, Idesis S, Escrichs A, Perl YS, et al. Off-Equilibrium Fluctuation-Dissipation Theorem Paves the Way in Alzheimer’s Disease Research [Internet]. 2024 [cited 2024 Oct 2]. Available from: http://biorxiv.org/lookup/doi/10.1101/2024.09.15.613131

43.	Comolatti R, Pigorini A, Casarotto S, Fecchio M, Faria G, Sarasso S, et al. A fast and general method to empirically estimate the complexity of brain responses to transcranial and intracranial stimulations. Brain Stimulat. 2019 Sep;12(5):1280–9.
